# Foreign children with cancer in Italy

**DOI:** 10.1186/1824-7288-37-44

**Published:** 2011-09-18

**Authors:** Roberto Rondelli, Giorgio Dini, Marisa De Rosa, Paola Quarello, Gianni Bisogno, Maurizio Aricò, Carivaldo Vasconcelos, Paolo Tamaro, Gabriella Casazza, Marco Zecca, Clementina De Laurentis, Fulvio Porta, Andrea Pession

**Affiliations:** 1 Paediatric Oncology-Haematology "Lalla Seràgnoli", Policlinico S.Orsola-Malpighi, Bologna, Italy; 2 Department of Paediatric Haematology and Oncology, Institute G.Gaslini, Genova, Italy; 3 Interuniversity Computing Centre (CINECA), Bologna, Italy; 4 Paediatric Onco-Haematology, Stem Cell Transplantation and Cellular Therapy Division, Regina Margherita Children's Hospital, Turin, Italy; 5 Department of Paediatrics, Division of Haematology Oncology, University Hospital of Padua, Padua, Italy; 6 Department of Paediatric Haematology-Oncology Azienda Ospedaliero Universitaria Meyer Children Hospital, Florence, Italy; 7 Institute for Maternal and Child Health, IRCCS "Burlo Garofolo", University of Trieste, Trieste, Italy; 8 Paediatric Haematology Oncology, Bone Marrow Transplant, Azienda Ospedaliero Universitaria Pisana, Ospedale S. Chiara, Pisa, Italy; 9 Paediatric Haematology/Oncology, Fondazione IRCCS Policlinico "San Matteo", Pavia, Italy; 10 Department of Paediatric Haematology/Oncology, IRCCS, Bambino Gesù Hospital, Rome, Italy; 11 AIEOP President, Oncology-Haematology and BMT Unit, Ospedale dei Bambini, Spedali Civili, Brescia, Italy

## Abstract

**Background:**

There has been a noticeable annual increase in the number of children coming to Italy for medical treatment, just like it has happened in the rest of the European Union. In Italy, the assistance to children suffering from cancer is assured by the current network of 54 centres members of the Italian Association of Paediatric Haematology and Oncology (AIEOP), which has kept records of all demographic and clinical data in the database of Mod.1.01 Registry since 1989.

**Methods:**

We used the information stored in the already mentioned database to assess the impact of immigration of foreign children with cancer on centres' activity, with the scope of drawing a map of the assistance to these cases.

**Results:**

Out of 14,738 cases recorded by all centres in the period from 1999 to 2008, 92.2% were born and resident in Italy, 4.1% (608) were born abroad and living abroad and 3.7% (538) were born abroad and living in Italy. Foreign children cases have increased over the years from 2.5% in 1999 to. 8.1% in 2008.

Most immigrant children came from Europe (65.7%), whereas patients who came from America, Asia and Oceania amounted to 13.2%, 10.1%, 0.2%, respectively. The immigrant survival rate was lower compared to that of children who were born in Italy. This is especially true for acute lymphoblastic leukaemia patients entered an AIEOP protocol, who showed a 10-years survival rate of 71.0% vs. 80.7% (p < 0.001) for immigrants and patients born in Italy, respectively.

**Conclusions:**

Children and adolescents are an increasingly important part of the immigration phenomenon, which occurs in many parts of the world. In Italy the vast majority of children affected by malignancies are treated in AIEOP centres. Since immigrant children are predominantly treated in northern Italy, these centres have developed a special expertise in treating immigrant patients, which is certainly very useful for the entire AIEOP network.

## Background

Although immigration in Italy is considered as a recent occurrence, our country has been traversed for centuries by different migration flows, which have always influenced our social and cultural life, and which is visible by the numerous ethnic and linguistic minorities still present today.

In the modern era, the phenomenon of immigration in Italy started around the 70s, but only in the early 80's it assumed such dimensions, so as to push the enacting of the first law for immigration regulation in 1986 [1]. In the early 90's, foreigners living in Italy were estimated in more than half a million, with less than 5% being children. According to the latest report of the Italian National Institute of Statistics (Istat), there were approximately 4 million foreigners living in Italy in 2008, and they represented 6.5% of the total population, with more than 20% (or 863,453) being children, over half of whom were born foreigners in our country [2].

However, in Italy the overall share of foreigners is lower than that of other European countries with older immigration history, ranking only above Finland and Portugal. Those numbers change considerably if taken into consideration only northern Italy, which become comparable to France's numbers [3].

Most foreigners living in Italy, according to the Istat report of 2009, come from Europe (53.6%), whereas Africa, Asia, America, and Oceania account for 22.4%, 15.8%, 7.9%, and 0.1% of Italy's immigrants, respectively.

### Foreign minor and immigrant

According to the Italian law, a foreigner is a non European Union (EU) citizen.

As reported in the Laws 268/1998 and 394/1999, a person who was born in Italy of non-citizen parents is only able to acquire the Italian citizenship after reaching the legal majority. Indeed, Italian citizenship is granted automatically only to children of Italian citizens [4,5].

If not born in Italy, foreign minors may enter our country to be reunited with a parent (or guardian), in response to a request for asylum, to be adopted, to study, to pursue professional sports, in solidarity programs, or for health reasons.

The entry in Italy of a foreign citizen for health reasons requires a request of a special visa, provided that the foreign national can financially support the treatment, individually or with the support of an association acknowledged by regional government, and after the approval from the chosen health facility.

However, most cases of foreign child treatment fall under the humanitarian aid provided by the Ministry of Health in cooperation with the Ministry of Foreign Affairs, which ensures full coverage of health services, in reciprocal agreements on health care [6,7]. Another source of financial support is represented by humanitarian programs adopted by the regions, authorizing local health authorities to provide highly specialized services for foreigners from countries that lack the necessary skills.

Moreover, even children who entered illegally in our country have all fundamental rights (including health care) guaranteed, in compliance with the New York Convention on the Rights of the Child of 1989, and as stipulated by the Law 189/2002 [8,9].

### Health care for foreign children with cancer: the AIEOP centres and the Registry Mod.1.01

Providing health care to foreign children put to a severe test the organizational abilities of our health care facilities, since it requires a whole new level of cultural and social awareness imposed by the continuous arising of the multicultural immigration.

In Italy, the assistance of all children with cancer is assured by the current network of 54 centres of the Italian Association of Paediatric Haematology and Oncology (AIEOP).

Since 1989, all data has been recorded in the database of AIEOP Mod.1.01 Registry [10,11]. This allows the monitoring of admissions, the evaluation of their biological and demographic characteristics and some indicators of quality of care, such as adherence to official protocols and survival.

Therefore, we wanted to use the database of Mod.1.01 Registry to assess the impact of immigration of foreign children with cancer on AIEOP centres' activity, in order to draw a map of assistance to these cases.

## Patients and methods

### Setting and sample

To this purpose, we used information on all registered cancer cases in the database of Mod.1.01 Registry from AIEOP centres, with age of less than 18 years old at diagnosis, from 1999 (the year in which began the systematic collection of the foreign country of birth and residence) to 2008.

We prefer the definition of immigrant to the definition of foreigner, since the former allows assessing the phenomenon more completely, as it also includes patients from EU countries whereas the second is simplistic and incomplete (as a synonym for non-EU citizen).

Therefore, the information relating to immigrant children (foreign-born and resident in Italy or abroad), was compared to that of cases born and resident in Italy. The cases born in Italy and living abroad were excluded from the study, because they were considered migrants.

### Statistical analysis

Data were analyzed as of December 31, 2009. All data were stored in a central database (Mod.1.01 Registry), and were processed at the AIEOP Operation Office [12]. Patients' characteristics, such as recruitment by year, type of disease, age at diagnosis, geographical area of origin, distribution on the Italian territory, and treatment centre, were analyzed and compared, when appropriate, using the χ^2 ^or Fisher's exact test in the case of discrete variables, or the Mann-Whitney test in the case of continuous variables. 95% Confidence Interval (CI) of mean or percentage were reported in case of statistically significant difference.

Overall survival (OS) was computed from the date of diagnosis to the date of death from any cause, or the last date of contact, if still alive. Survival distributions were estimated using the method of Kaplan and Meier, and the log-rank test was used to examine differences among subgroups [13]. Results were expressed as cumulative probability (%) and standard error (SE).

Since the follow-up numbers were based only on contact to AIEOP centers - available only in 47% of all cases, being 43% immigrants and 47% born in Italy, - and not supplemented by active research, we chose to report OS only for cases affected by acute lymphoblastic leukaemia (ALL) entered an AIEOP protocol, as the follow-up data was available for 93% of ALL cases. Unfortunately, these follow-up data were missing from 35% to 75% of cases affected by other disease and for the majority of cases enrolled in an unofficial protocol. Therefore, we had to leave such cases out of our study.

Multivariate Cox proportional hazards analysis was used to explore some basic factors potentially associated with survival in ALL treaded with an AIEOP protocol: patient's birth place (immigrants vs. born in Italy), patients' gender (male vs. female), age at diagnosis (> 14 years vs. ≤ 14 years) and risk group (high vs. no-high) [14].

Adequacy of data to the proportional hazard assumptions of the Cox model was verified by plotting the logarithm of the cumulative hazard function against the logarithm of survival time, checking for parallelism.

All *P *values are 2-sided and values less than 0.05 were considered statistically significant.

Statistical analysis was carried out using the Stata statistical package, version 7.0 [15].

## Results

For 14,738 cases out of 14,868 (99.1%) recorded by all AIEOP centres in the period from 1999 to 2008 from, in which the place of birth and residence were known, excluding 27 cases born in Italy but emigrated abroad, 92.2% (13,592 cases) were born and resident in Italy, while 4.1% (608) were born and living abroad, and 3.7% (538) were born abroad and living in Italy. Excluding 293 cases that were born in one of the 27 EU countries, non-EU amounted to 5.8% of the cases.

**Figure 1:**
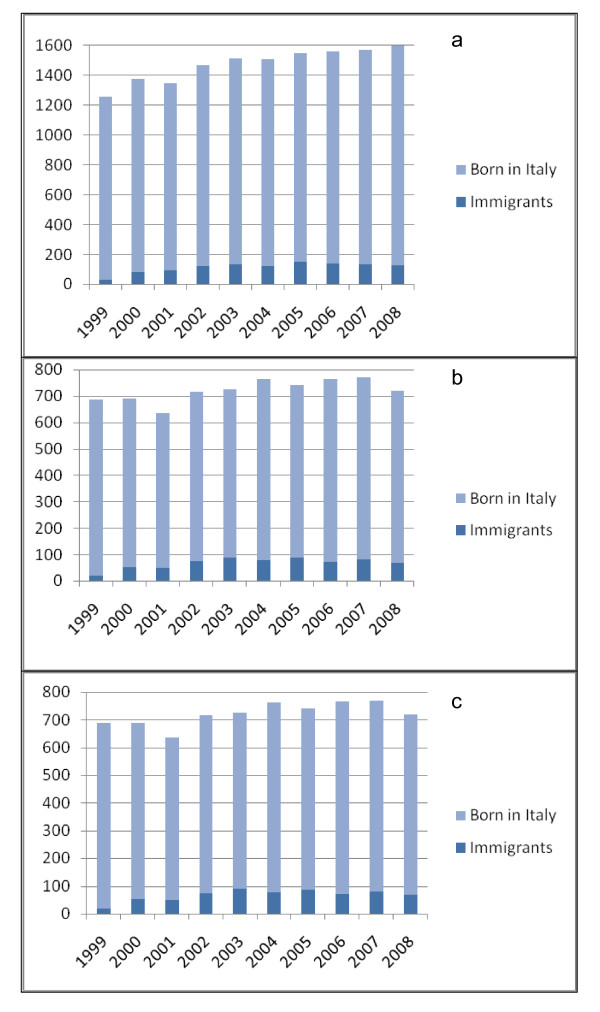
**Overall accrual by year (a), for leukaemia and lymphoma (b) and solid tumours (c)**.

The admission of immigrant children has gradually and steadily increased over the years, from 31 cases, 2.5% of the total in 1999 to 130 cases, 8.1% of the total in 2008, with an average of about 115 cases/year, while cases born in Italy amounted to about 1,360 cases/year (Figure 1).

Foreign children amount to 9.3% of cases with leukaemia and lymphoma, and 6.3% of cases with solid tumours. The number of immigrant cases with leukaemia and lymphoma increased over the years from 2.8% in 1999 to 9.4% in 2008, while solid tumours increased from 2.1% to 7.1% in the same period (Figure 1).

Most immigrant children seeking treatment come from Europe (65.7%); 40.1% from outside the EU, of which the majority from Albania (21.5%), from the countries of the former Yugoslavia (10.9%), Ukraine (4.3%) and Russia (1.0%) and 25.6% from EU countries such as Romania (16.8%), Germany (2.1%) and Greece (1.7%), while only 6 cases are born and resident in the Republic of San Marino.

**Figure 2:**
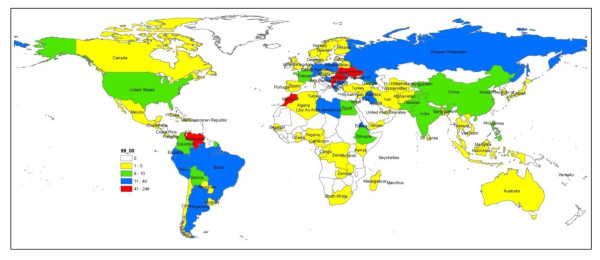
**Migration flows by country of origin**.

The 13.2% of immigrants in need of medical care came from the Americas, led by Venezuela (4%), followed by Ecuador (1.9%); 10.8% from Africa, mainly from Morocco (3.8%) and Libya (1.6%); 10.1% from Asia, especially from Iraq (1.4%), and only 2 cases from Oceania (0.2%) (Figure 2).

**Figure 3:**
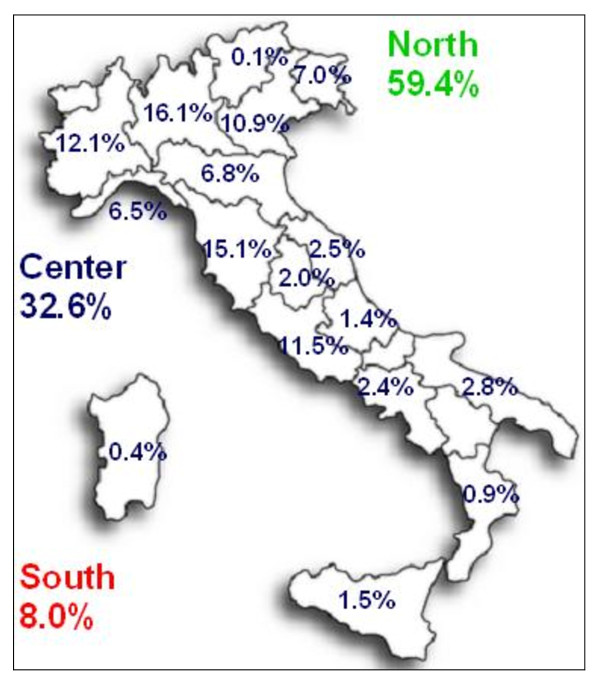
**Percentage of immigrant cases by geographical area and region of treatment**.

The 59.4% of immigrant children cases were being treated in AIEOP centres of northern Italy, 32.6% in the centres of central Italy, while only 8% in southern Italy centres, whereas for cases born in Italy, the distribution was: 54.1%, 22.5% and 23.4%, respectively (Figure 3).

The male/female ratio was slightly higher for immigrants than for cases of patients who were born in Italy (1.4 vs. 1.3). It was also true regarding the age: 8.0 (95% CI 7.7-8.2) years old vs. 6.7 (95% CI 6.6-6.8) years old in those cases of patients born in Italy (p < 0.001), which resulted in a greater proportion of adolescents (> 10 years) among immigrants (41% vs. 32% of patients born in Italy).

About 59% (95% CI 56-62) of immigrant cases consisted of leukaemia and lymphoma vs. 48% (95% CI 47-49) of cases born in Italy (p < 0.001).

The frequency of leukaemia was greater for immigrants (46.9% vs. 33.4%), in which prevails ALL, but also with high relative frequency of acute non-lymphoblastic leukaemia and other leukaemia, mainly chronic myeloid leukaemia, of which immigrant children represented over 15% of all cases of these two forms registered by AIEOP centres. The frequency of lymphomas was rather similar in both groups (Table 1).

**Table 1:** Distribution of cases by diagnosis

Diagnosis	Immigrants		Born in Italy		Total	
	N.cases	%	N.cases	%	N.cases	%
**Leukaemia**	**537**	**46.9**	**4,536**	**33.4**	**5,073**	**34.4**
*ALL*	*347*	*30.3*	*3,593*	*26.4*	*3,940*	*26.7*
*AML*	*132*	*11.5*	*710*	*5.2*	*842*	*5.7*
*OL*	*58*	*5.1*	*233*	*1.7*	*291*	*2.0*
**Lymphoma**	**136**	**11.9**	**2,008**	**14.8**	**2,144**	**14.5**
*HL*	*49*	*4.3*	*1,047*	*7.7*	*1,096*	*7.4*
*NHL*	*87*	*7.6*	*946*	*7.0*	*1,033*	*7.0*
**CNS tumours**	**119**	**10.4**	**2,119**	**15.6**	**2,238**	**15.2**
**SNS tumours**	**57**	**5.0**	**1,093**	**8.0**	**1,150**	**7.8**
*NB*	*55*	*4.8*	*1,061*	*7.8*	*1,116*	*7.6*
**RTB**	**39**	**3.4**	**308**	**2.3**	**347**	**2.4**
**Kidney tumours**	**26**	**2.3**	**621**	**4.6**	**647**	**4.4**
*WT*	*25*	*2.2*	*540*	*4.0*	*565*	*3.8*
**Liver tumours**	**11**	**1.0**	**128**	**0.9**	**139**	**0.9**
**Bone tumours**	**93**	**8.1**	**582**	**4.3**	**675**	**4.6**
*Osteorarcoma*	*48*	*4.2*	*231*	*1.7*	*279*	*1.9*
*ES*	*45*	*3.9*	*327*	*2.4*	*372*	*2.5*
**STS**	**68**	**5.9**	**885**	**6.5**	**953**	**6.5**
*RMS*	*41*	*3.6*	*438*	*3.2*	*479*	*3.3*
**GCT**	**10**	**0.9**	**382**	**2.8**	**392**	**2.7**
**Carcinomas**	**16**	**1.4**	**174**	**1.3**	**190**	**1.3**
*Thyroid*	*2*	*0.2*	*36*	*0.3*	*38*	*0.3*
*Melanoma*	*0*	*-*	*34*	*0.3*	*34*	*0.2*
**Other tumours**	**34**	**3.0**	**756**	**5.6**	**790**	**5.4**
***Total***	**1,146**	**100**	**13,592**	**100**	**14,738**	**100**

Legend: ALL, acute lymphoblastic leukaemia; AML, acute myeloblastic leukaemia; OL, other leukaemia; HL, Hodgkin's lymphoma; NHL, non-Hodgkin's lymphoma; CNS, central nervous system; SNS, sympathetic nervous system; NB, neuroblastoma; RTB, retinoblastoma; WT, Wilm's tumour; ES, Ewing's sarcoma; STS, soft tissue sarcoma; RMS, rabdomiosarcoma; GCT, germinal cells tumour.

Approximately 41% (95% CI 38-44) of immigrant cases consisted of solid tumours vs. 52% (95% CI 51-53) of cases from patients born in Italy (p < 0.001). The relative frequency of solid tumours was lower in immigrants, except those of retinoblastoma (3.4% vs. 2.3%), where immigrant children represented over 10% of all cases recorded by the AIEOP centres with this type of cancer (Table 1).

It is important to highlight that the relative frequency of bone tumours in immigrants was two times higher than that of patients born in Italy, especially osteosarcoma, which as many as 17% of all cases are immigrants (Table 1). Most of these patients resulted born in Romania (31.3%), Albania (27.1%) and Libya (10.4%).

Finally, low was the incidence of germ cell tumours in immigrants, while the relative frequency of carcinomas was similar in both groups (Table 1).

The number of immigrant cases treated with AIEOP protocols was significantly lower than that of cases of patients born in Italy: 56% (95% CI 53-59) vs. 73% (95% CI 72-74), p < 0.001. Same result if we consider the cases with leukaemia and lymphoma: 60% (95% CI 56-64) vs. 90% (95% CI 89-91), p < 0.001, or solid tumours: 51% (95% CI 46-55) vs. 57% (95% CI 56-58), p < 0.01.

Only 6,896 cases out of 14,738 (46.8%) resulted evaluable for survival analysis: 5,422 were alive (354 immigrants and 5,068 born in Italy) and 1,474 were deceased (168 immigrants and 1,306 born in Italy), while the number of lost to follow-up were 56 (0.8%): 7 immigrants and 49 born in Italy.

After a median observation time similar in both groups (48 months for immigrants vs. 52 months for born in Italy) the survival of immigrant cases was significantly lower (p < 0.001) compared to that of patients who were born in Italy: 10-years OS: 53.2% (SE 4.4) vs. 70.8% (SE 1.3).

**Figure 4:**
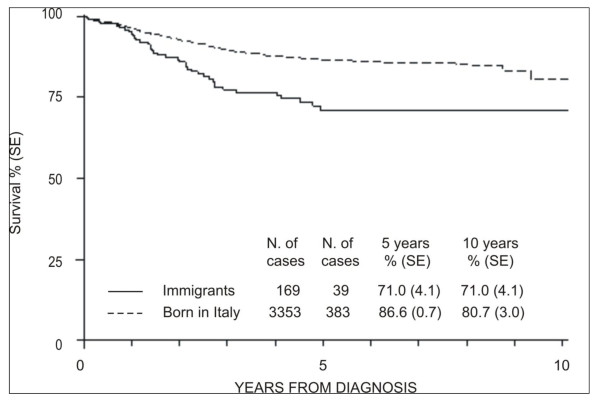
**Overall survival for acute lymphoblastic leukaemia cases entered an AIEOP protocol**.

In ALL patients treated by an AIEOP protocol, after a median observation time similar in both groups (54 months for immigrants vs. 55 months for born in Italy) the survival of immigrant cases was significantly lower (p < 0.001) compared to patients who were born in Italy: 10-years OS: 71.0% (SE 4.1) vs. 80.7% (SE 3.0) (Figure 4).

Always in this group of patients, analysis of prognostic factors, also underscored that being an immigrant was a negative and independent prognostic factor with risk of death of more than 1.5 times compared to cases of patients who were born in Italy, likewise high risk group, male and age > 14 years (Table 2).

**Table 2:** Survival: multivariate analysis in acute lymphoblastic leukaemia cases entered an AIEOP protocol

Variables	Hazard Ratio (95% Confidence Interval)	p
Age > 14 years vs. Age ≤ 14 years	2.20 (1.51 - 3.21)	0.000
Male vs. Female	1.49 (1.20 - 1.86)	0.000
High risk vs. no-High risk	2.01 (1.28 - 3.16)	0.002
Immigrants vs. Born in Italy	1.70 (1.16 - 2.50)	0.007

## Discussion

Migration has always involved an uncountable number of motivations and reasons, and it has marked the history of mankind over the centuries. Moreover, it is no surprise that nowadays children and adolescents are increasingly being perceived as an important part of the immigration phenomenon.

The entry of several Eastern European countries into the EU, and today's rapid dissemination of information, have produced in the last ten years an acute increase in families from developing countries coming to Western European countries seeking the best possible treatment for children suffering from cancer, and Italy is no exception.

The analysis of the database of Mod.1.01 Registry showed that immigrants represented about 8% of all cases registered by AIEOP centres, totalling approximately 115 cases/year (85 from non-EU countries).

The geographical origin of immigrant cases was similar, though not identical, to that reported by the Istat. Among the foreign cases from European countries admitted in AIEOP centres, for example, despite the Istat reporting a rapid increase in the Romanian community (currently the main foreign community in our country), Albanians still exceeded the Romanians in the AIEOP statistics. Among the Asian nationalities, Iraqis were more numerous than the Chinese, while the Venezuelans were more than the Ecuadorians among American nationalities. Finally, patients from Morocco were confirmed to be the most represented among the Africans, as also reported by the Istat [2]. However, it is possible that the high number of cases from Iraq and Venezuela were largely influenced by programs of health cooperation between Italy - through the Ministry of Health or some Italian regions, - and those countries, in accordance with the provisions of the guidelines of the Italian development cooperation [16,17].

We have already mentioned how immigrant children are accepted predominantly (59%) by AIEOP centres of northern Italy, the same as for cases of patients who were born in Italy [18].

At the same time, AIEOP centres of central Italy accepted 33% of immigrants and the proportion of these immigrant cases on overall treated cases was considerably high (15% in Tuscany and Umbria), even if the higher proportion was found in the northern region of Friuli (20%).

The centres of these regions (northern and central Italy) have therefore developed special expertise and skills in treating immigrant patients, which are certainly very useful for the AIEOP network.

Ultimately, as opposed to the cases of patients who were born in Italy, we had seen a higher number of males in immigrant children, a higher age at diagnosis, and a greater and significant share of leukaemia and lymphoma, especially those of more severe prognosis types. That was probably due to the fact that most of the cases that came to Italy were only to perform haematopoietic stem cell transplantation (HSCT), which may explain in part the lower survival, together with the reduced share of cases that were treated with AIEOP protocols.

It is also important noticing that the higher relative percentage of immigrants affected and treated by retinoblastoma or osteosarcoma, even if not that significant, were probably due to the high specialization needed to treat this disease, which probably may have led the immigrants to contact some specialized AIEOP center with international know-how.

There are several possible causes for recruitment failure in AIEOP protocols, and they can be rather difficult to explain: on one hand, 23% of these cases had their diagnosis made in the country of origin, and they were pre-treated with other protocols, which undermines AIEOP protocols eligibility. On the other hand, cases may be in a stage of disease that only allows individualized therapy or HSCT. In fact, although 31% of immigrants were submitted to HSCT, in 17% of these cases, the patient arrived in Italy just to perform transplantation.

We found a significant lower OS in immigrants compared to the cases of patients who were born in Italy, but follow-up data was available only for 47% of all cases, which could definitely mislead the final statistics.

Therefore, we decided to consider only the group of ALL cases entered an AIEOP protocol, in which follow-up was updated for all cases, and which would consequently be a more reliable source of data. Using such criteria, the OS was similar to that reported by AIEOP in last long-term results report on study 95: 10-years OS 80.3% (1.7) vs. 82.4% (SE 1.0) [19].

The significant difference in terms of OS reported between immigrants with ALL compared to ALL in patients who were born in Italy was probably due in part to a higher and significant rate of high risk cases in immigrants (17.9%, 95% CI 12-24, vs. 2.5%, 95% CI 2-3, p < 0.001) and in part to other yet undefined factors. In addition, to be an immigrant seemed to play a significant and independent role affecting OS, such as high risk group, age > 14 years and male gender, but this need more detailed studies including all known prognostic factors in the case of ALL.

## Conclusions

Undeniably, this analysis has highlighted some limitations of the Mod.1.01 Registry, such as the fact that it was not possible to identify children born in Italy to foreign parents.

Another limitation was the difficulty in obtaining a homogeneous follow-up of all cases, but for patients entered in some AIEOP protocol, such as for ALL, which data base is linked to Mod.1.01 Registry data base. Despite these limitations, survival analysis has demonstrated the poorer prognosis for immigrants children compared to patients who were born in Italy, which could depend on some known (i.e. higher risk cases) and unknown factors that could be investigated with "ad hoc" studies.

Nonetheless, Mod.1.01 Registry has demonstrated its effectiveness as an instrument to measure the phenomenon of immigration of foreign-born children suffering from cancer, who referred to the network of AIEOP centres, and also to identify the new needs of the paediatric population with cancer in Italy, which tends to be increasingly more multiracial in the near future.

Indeed, cultural, religious, and social differences deeply affect the way patients and their families face the disease - besides the limitations on communication due to linguistic barriers, - and as a result, it is currently required organizational and administrative improvements on how to assist foreign patients with cancer.

In addition, the diverse origin of those patients reports a greater need to provide adequate documentation to the families coming to the AIEOP centres, information concerning the ward's organization and rules, and informed consent in the patients' languages, as well as the need for cultural mediators to communicate with family members not only at critical moments, but often daily, during regular therapeutic procedures.

## Competing interests

The authors declare that they have no competing interests.

## Authors' contributions

RR, GD, FP and AP were involved in the conception and design of the study. MDR carried out data extraction. RR conducted statistical analysis. RR, GD and AP drafted the paper with contributions from the co-authors, and CV also performed the linguistic revision of the manuscript. All authors have read and approved the final version of the manuscript.
